# Stearoyl-CoA desaturase 1 deficiency drives saturated lipid accumulation and increases liver and plasma acylcarnitines

**DOI:** 10.1016/j.jlr.2025.100824

**Published:** 2025-05-09

**Authors:** Mugagga Kalyesubula, Helaina Von Bank, Jessica W. Davidson, Maggie S. Burhans, Madelaine M. Becker, Ahmed Aljohani, Judith Simcox, James M. Ntambi

**Affiliations:** 1 Department of Biochemistry, University of Wisconsin-Madison, Madison, WI, USA; 2 Department of Nutritional Sciences, University of Wisconsin-Madison, Madison, WI, USA; 3 College of Science and Health Professions, King Saud Bin Abdulaziz University for Health Sciences, Riyadh, Saudi Arabia; 4 King Abdullah International Medical Research Center (KAIMRC), Riyadh, Saudi Arabia; 5 HHMI, Department of Biochemistry, University of Wisconsin-Madison, Madison, WI, USA

**Keywords:** SCD1, SCD5, saturated fatty acid, oleate, acylcarnitines, lipidomics

## Abstract

Stearoyl-CoA desaturase-1 (SCD1) is a critical regulator of lipogenesis that catalyzes the synthesis of MUFAs, mainly oleate (18:1n-9) and palmitoleate (16:1n-7) from saturated fatty acids, stearoyl-CoA (18:0) and palmitoyl-CoA (16:0), respectively. Elevated SCD1 expression and its products are associated with obesity, metabolic dysfunction-associated steatotic liver disease, insulin resistance, and cancer. Conversely, *Scd1* deficiency diminishes de novo lipogenesis and protects mice against adiposity, hepatic steatosis, and hyperglycemia. Yet, the comprehensive impact of *Scd1* deficiency on hepatic and circulating lipids remains incompletely understood. To further delineate the effects of SCD1 on lipid metabolism, we employed lipidomics on the liver from mice under a lipogenic high carbohydrate, very low-fat diet. We found that *Scd1* deficiency leads to an accumulation of saturated lipids and an increase in hepatic and plasma acylcarnitines. Remarkably, transgenic replenishment of de novo oleate synthesis by human *SCD5* in the liver of *Scd1*-deficient mice not only restored hepatic lipid desaturation levels but also attenuated acylcarnitine accumulation, highlighting the distinct role of SCD1 and oleate in regulating intracellular lipid homeostasis.

Stearoyl-CoA desaturase (SCD) is a crucial lipogenic enzyme anchored in the endoplasmic reticulum, which catalyzes the delta-9 cis desaturation of saturated fatty acids into MUFAs, mainly palmitate and stearate into palmitoleate and oleate, respectively (1, 2). The MUFAs, in turn, are key substrates for the biosynthesis of complex lipid molecules, such as triglycerides, phospholipids, and wax esters, involved in various cellular processes (3, 4). SCD exists as multiple isoforms, with humans expressing SCD1 and SCD5 and mice expressing SCD 1–4 (2, 5). SCD1 is the predominant isoform and exhibits a high degree of sequence homology (>87%) among human, mouse, cattle, and chicken proteins, suggesting an evolutionarily conserved function of SCD1 in regulating lipid metabolism (5, 6).

The role of SCD1 in synthesizing MUFAs to promote intracellular homeostasis places it as one of the critical metabolic hubs that coordinates metabolic pathways in glycolipid metabolism, insulin signaling, and inflammation (2, 6). It is transcriptionally regulated by various hormonal, nutritional, and environmental factors, such as fatty acids, glucose, and leptin (6, 7, 8, 9, 10). However, overexpression of *SCD1* is implicated in various metabolic disorders, including obesity, metabolic dysfunction-associated steatotic liver disease (MASLD), insulin resistance, and cancer (11, 12, 13, 14). This is important as MASLD is the leading cause of chronic liver disease complications, such as cirrhosis and liver cancer (15). Moreover, human genetic studies linked *SCD1* gene polymorphisms to variations in insulin sensitivity and body fat distribution (16, 17). Therefore, as a hub gene influencing various metabolic outcomes, targeting SCD1 therapeutically is an attractive option to address multifaceted metabolic diseases, such as MASLD, cancer, and diabetes (6, 18, 19, 20). Thus, studies that improve our understanding of the physiological role of SCD1 and the metabolic implications of its inhibition are crucial.

Our previous studies utilizing various models have shown the role of SCD under multiple metabolic conditions, including obesity, steatosis, and hyperglycemia (1, 21). We demonstrated that *Scd1* deficiency modulates glucose metabolism, enhancing glucose uptake through the augmentation of *Glut1*, *Glut4*, and *Fgf21* (22). Notably, *Scd1* deficiency has been shown to protect against obesity and fatty liver development. This is likely through the reduction of hepatic de novo fat synthesis by downregulating the expression of crucial genes, such as sterol regulatory element binding transcription factor 1 (*Srebp1*), carbohydrate response element binding protein (*Chrebp*), fatty acid synthase (*Fasn*), and acetyl-CoA carboxylase alpha (*Acaca*) (1, 23). However, the broad impact of SCD1 on the hepatic lipidome and subsequent alterations in circulating lipid levels remain to be fully determined.

In the current study, we performed targeted and untargeted lipidomic approaches to determine the impact of *Scd1* deficiency, revealing substantial changes in lipid levels and increased lipid saturation in both the liver and plasma. Intriguingly, *Scd1* deficiency elevated hepatic and plasma acylcarnitine levels. We hypothesized that oleate, a product of SCD1, could counteract these alterations. Indeed, replenishment of endogenous oleate by the transgenic expression of human *SCD5* in the livers of *Scd1*-deficient mice restored lipid desaturation and lowered acylcarnitines. These findings highlight a previously unappreciated role of SCD1 in modulating acylcarnitine metabolism.

## Materials and Methods

### Animals and diets

All studies involving animals were approved and conducted in accordance with the Institutional Animal Care and Use Committee guidelines of the University of Wisconsin-Madison (protocol #A005125). The mice were taken care of by the University of Wisconsin-Madison animal care facility with free access to food and water on a regular 12-h light/dark cycle. All the mice employed in this study were in the C57BL/6 genetic background and were offered a standard rodent chow diet (Purina 5008; Harlan Teklad, Madison, WI) until being changed to experimental diets. At ∼8–10 weeks, the mice were shifted into individual cages and fed a high carbohydrate very low-fat diet (HCD; Harlan Teklad; TD.03045) for 10–14 days. Generation of *Scd1*^*−/−*^ (*Scd1* global KO [GKO]), *SCD5*Tg+; *Scd1*−/− (GKO mice expressing human *SCD5* in the liver [TG5]), *Scd1*^*lox/lox*^ (*Scd1* LOX control), and *Scd1*^*lox/lox*^; Albumin Cre/+ (liver-specific SCD1 KO [LKO]) mice has been previously described (1, 21, 24). All mice were euthanized by an isoflurane overdose after a 4-h fasting, and harvested tissues and plasma were snap-frozen under liquid nitrogen and stored under −80°C.

### RNA isolation and real-time quantitative PCR

RNA was harvested from the liver using Trizol reagent (Life Technologies/Invitrogen, Carlsbad, CA), homogenized with a TissueLyzer II (Qiagen), and treated with Turbo DNase (Ambion). cDNA from the RNA was synthesized using a High-Capacity cDNA Reverse Transcription Kit (Applied Biosystems). Real-time quantitative PCR analysis was carried out using PowerUp SYBR Green 2x Master Mix (ThermoFisher Scientific) in a 384-well plate on an Applied Biosystems QuantStudio 5 Real-Time PCR System. Relative gene expression was determined by employing the ΔΔCT method (25), normalized to the expression of *Rps3* as a housekeeping gene. A list of primer pairs is included in supplemental Table S1.

### Western blotting

An aliquot of frozen liver sample was homogenized in ice-cold RIPA buffer (Boston Bioproducts) containing a protease inhibitor (ThermoFisher Scientific) using a tissue lyzer (Quiagen, Hilden, Germany). The lysate was centrifuged at 14,000 rpm at 4°C for 15 min, and the protein amount was quantified by a BCA assay (ThermoFisher Scientific). For immunoblot assessment, 20 μg of protein was diluted in Laemmli loading buffer (Bio-Rad), heated for 5 min at 90°C, and run on a 4–20% acrylamide gel (ThermoFisher Scientific), and transferred to a nitrocellulose membrane. Blots were visualized by chemiluminescence using an iBright FL1500 machine (ThermoFisher Scientific). The employed antibodies against HNF4α (MA1199), CPT2 (MA532314), and β-ACTIN (PA1183) were purchased from ThermoFisher Scientific. ImageJ software (National Institutes of Health, Bethesda, MD) was utilized for densitometric quantification.

### Lipidomics

All the reagents employed were HPLC grade or higher. Water (catalog no.: LC365), methanol (catalog no.: LC230), acetonitrile (ACN; catalog no.: LC015), and ammonium formate (catalog no.: 55674) were purchased from Honeywell; ethyl acetate (catalog no.: 650528) and butylated hydroxytoluene (catalog no.: B1378) were purchased from Sigma; and isopropanol (IPA; catalog no.: A461) and formic acid (catalog no.: A11710X1) were purchased from Fisher Scientific.

Lipids were extracted with a mix of IPA, water, and ethyl acetate (3:1:6) with 0.01% butylated hydroxytoluene and added internal standards. For untargeted lipidomics, the internal standards were SPLASH Lipidomix II (Avanti; lot #6453WAA011, 10 μL/sample), oleoyl-L-carnitine (Levocarnitine) d3 (Cayman; #26578, 2.33 pmol/sample), and stearic acid d35 (Cayman; #9003318, 90 nmol/sample). For targeted acylcarnitine assessment, the NSK-B acylcarnitine mix was added (Cambridge Isotopes, 5 μL/sample). Briefly, 500 μL of extraction mix was added to plasma (25 μL for untargeted, 50 μL for acylcarnitine) or liver (19–22 mg for untargeted, 22–27 mg for acylcarnitine) in ceramic bead tubes (Omni Int; #19-627). Samples were shaken in a TissueLyzer (frequency 30/s, 30 s) until fully homogenized, with a break (5 min, 4°C) every two cycles. Lysates were incubated at −20°C for 10 min and then centrifuged (16,000 *g*, 4°C, 10 min). The supernatant (425 μL) was transferred to a microcentrifuge tube, the spin was repeated, and the subsequent supernatant (350 μL) was transferred to a new tube. Extracts were evaporated by vacuum (40°C, 2 h).

Lipidomics was performed by ultra-HPLC (Agilent 1290 Infinity II BioLC) on a ZORBAX C18 column (Agilent; #959758-902) followed by triple quadrupole mass spectrometry (Agilent; 6495C) using the chromatography gradient and dynamic multiple reaction monitoring (dMRM) settings outlined (26) (Agilent Application Note #5994-3747EN). Dried lipid extracts were resuspended in IPA (150 μL), transferred to injection vials with glass inserts (Agilent; #5183-2086), and capped (Agilent; #5185-5820). Liver samples were diluted 1:8 in IPA. Samples were maintained at 21°C in the multisampler, and 1 μL injections were run in a randomized order. Pooled matrix samples were used to update retention times from the published protocol. Peak integration was validated based on available data of isotope patterns; lysophospholipids with two resolved peaks were annotated “a” or “b.” Peaks were smoothed via the Quadratic/Cubic Savitzky-Golay method and then normalized to the relative peak abundance of a representative internal standard using MassHunter Quantitative Analysis (Agilent). Peaks that had an equal or higher abundance in a no-sample blank were removed. Raw data files were converted to mzML and uploaded to MassIVE (#MSV000096321).

Targeted lipidomic analysis of acylcarnitines was then performed on the livers and plasma of LKO mice. Dried lipid extracts were resuspended in methanol (150 μL) and diluted 1:5 for analysis. Acylcarnitines were measured by liquid chromatography (Agilent 1290 Infinity II) coupled to a triple quadrupole mass spectrometer (Agilent 6495). Injection volumes were 2 and 3 μL for liver and plasma, respectively. Extracted lipids were separated on an Acquity BEH C18 column (Waters 186009453, 1.7 mm 2.1 × 100 mm) with a VanGuard BEH C18 precolumn (Waters; 18003975), maintained at 60°C. The chromatography gradient, outlined in supplemental Table S2, was composed of mobile phase A (60:40 ACN:H_2_O, 10 mM ammonium formate, and 0.1% formic acid) and mobile phase B (9:1:90 ACN/H_2_O/IPA, 10 mM ammonium formate, and 0.1% formic acid). Acylcarnitines were analyzed by dMRM with a cycle time of 500 ms (supplemental Table S3). Gas temperature was kept at 290°C with flow at 12 L/min. The nebulizer was set to 35 psig, the sheath gas temperature to 300°C, and the sheath gas flow at 11 L/min. Samples were run in randomized order with methanol injections spaced in between. Raw data were collected in .d format and manually inspected for retention time shifts. Peaks were normalized to a representative internal standard peak area. Lipid quantities were normalized to plasma volume or liver weight. Tables of parameters for liquid chromatography gradient for acylcarnitine analysis and parameters for dMRM analysis of acylcarnitines can be found in the (supplemental Tables S2 and S3).

### Chromatin immunoprecipitation sequencing

Binding tracks were obtained from HNF4α chromatin immunoprecipitation sequencing (ChIP-Seq) of livers from wild-type male C57BL/6J mice, as described (27). Briefly, chromatin was immunoprecipitated with the anti-hepatocyte nuclear factor 4 alpha (HNF4α) antibody (Abcam; ab181604), cleaned (Zymo; D5205), and sequenced with paired-end 150 bp sequencing (University of Wisconsin-Madison Biotechnology Center). After data quality control, sequence reads were aligned to the mouse genome (UCSC build mm10). Peaks for genes of interest were manually called from BigWig files using Integrative Genomics Viewer http://maq.sourceforge.net/.

### Statistical analysis

Statistical analyses of untargeted lipidomic data were conducted in MetaboAnalyst 6.0 (28). Unless otherwise stated, lipids were considered significant if they had a ≥2-fold change and a false discovery rate (FDR)-adjusted *P* value of <0.1. Other statistical analyses were performed in Jmp (version 17.0.0; SAS Institute, Inc, Cary, NC, 2016). Results are presented as mean ± SD. Comparisons were performed using an unpaired, two-tailed Student’s *t-*test. Untargeted lipidomics data cleanup, as well as Pearson correlations to determine associations between hepatic and plasma acylcarnitines, was conducted in R (version 4.3.2). Code can be found on GitHub (hcvonbank/Kalyesubula). Figures were generated using GraphPad Prism, version 10.4.1 (GraphPad Software, LLC, San Diego, CA). A significance threshold level of *P <* 0.05 was considered.

## Results

### *Scd1* deficiency alters the hepatic lipidome and increases saturated liver lipids

**Fig. 1:**
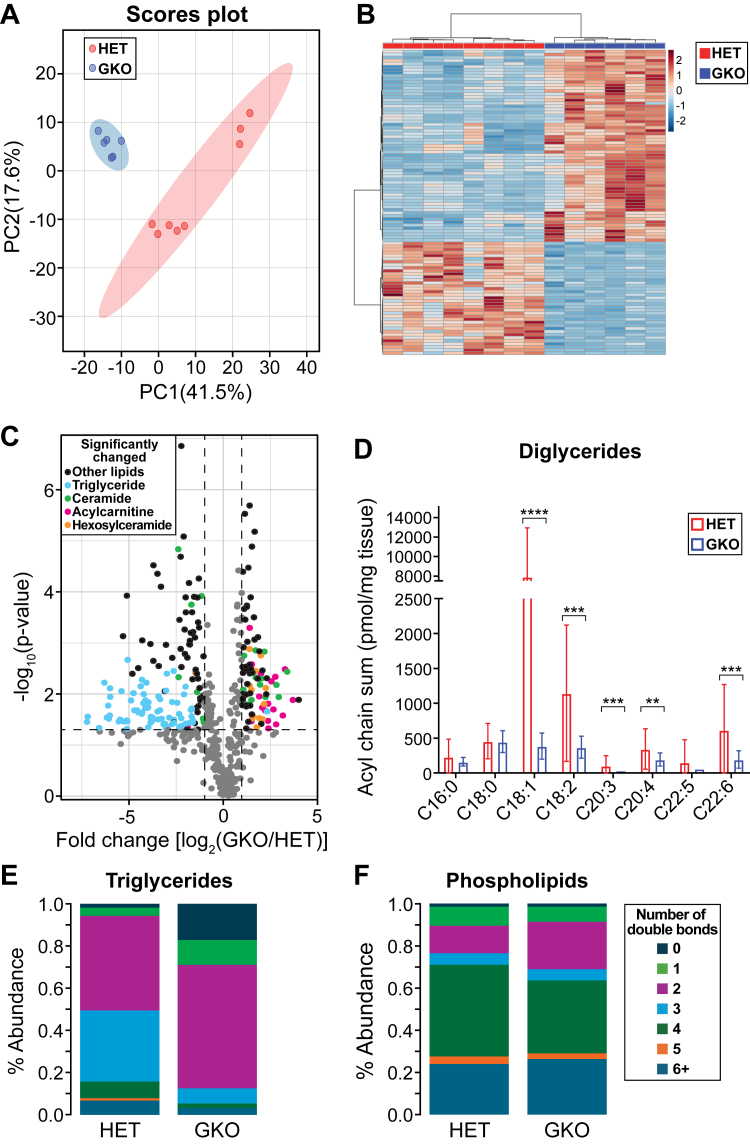
Global *Scd1* deficiency altered the hepatic lipidome and increased saturated liver lipids under an HCD. A: PCA of lipids between GKO and HET mice. B: Heatmap and cluster analysis of hepatic lipids. C: Volcano plot showing the significance and fold change between GKO and HET mice of liver lipids. The significance of the lipids was considered at an FDR-corrected *P* value of 0.1 and a fold change of 2. Dots on the right denote lipid increase in the GKO mice, whereas dots on the left denote lipid decrease in the GKO mice. D: Acyl chain sum of the diglycerides. Relative abundance for each degree of total desaturation (number of double bonds) in E: Triglycerides and F: Phospholipids. ∗*P <* 0.05, ∗∗*P <* 0.01, *∗∗∗P <* 0.001, and *∗∗∗∗P <* 0.0001. Data are presented as mean + SD. HET, heterozygote mice. N = 6–8 mice.

To determine the effect of global *Scd1* deficiency on the hepatic lipid profile of mice fed a lipogenic HCD diet, common in several parts of the world (29, 30), we conducted an untargeted lipidomic analysis using LC-MS. We employed global *Scd1*-deficient (GKO) mice and heterozygote mice as controls since the heterozygote mice phenocopied wild-type mice. The mice clustered according to their genotypes, as shown by a principal component analysis (PCA) plot and a heatmap (Fig. 1A, B). Over 500 lipids were commonly identified in the livers of the two groups of mouse genotypes. Strikingly, *Scd1* deficiency altered 223 lipids, with 87 elevated and 136 reduced (Fig. 1C and supplemental Table S4). The lipidomic signature of *Scd1* deficiency was characterized by increased levels of acylcarnitines, phospholipids, cholesteryl esters, ceramides, and hexosylceramides (Fig. 1C and Supplemental Fig. S1C). On the other hand, *Scd1* deficiency led to a decrease of several lipid species, including diglycerides, triglycerides, lysophospholipids, and free fatty acids (Fig. 1C and Supplemental Fig. S1C). These findings highlight the critical role of *Scd1* in regulating hepatic lipid metabolism.

To gain insights into the saturation levels of liver lipids after *Scd1* ablation, we analyzed the acyl chain distribution of diglycerides, which are precursors to triglycerides. Our analysis revealed a reduction in diglycerides containing a C18:1 fatty acyl chain in the GKO mice (Fig. 1D). We further assessed the double bond distribution in triglycerides, the primary form of fat storage. Triglyceride abundances for each degree of saturation were summed up, and then graphed as a relative abundance, uncovering a striking increase in fully saturated triglycerides in GKO mice (∼17%) compared with control mice (∼2%) (Fig. 1E). However, the levels of saturated phospholipids were similar between the two genotypes, approximately 1% (Fig. 1F). These findings underscore the crucial role of SCD1 in regulating saturation, particularly in glycerolipids.

### *Scd1* deficiency alters plasma lipids and increases the levels of saturated circulating lipids

**Fig. 2:**
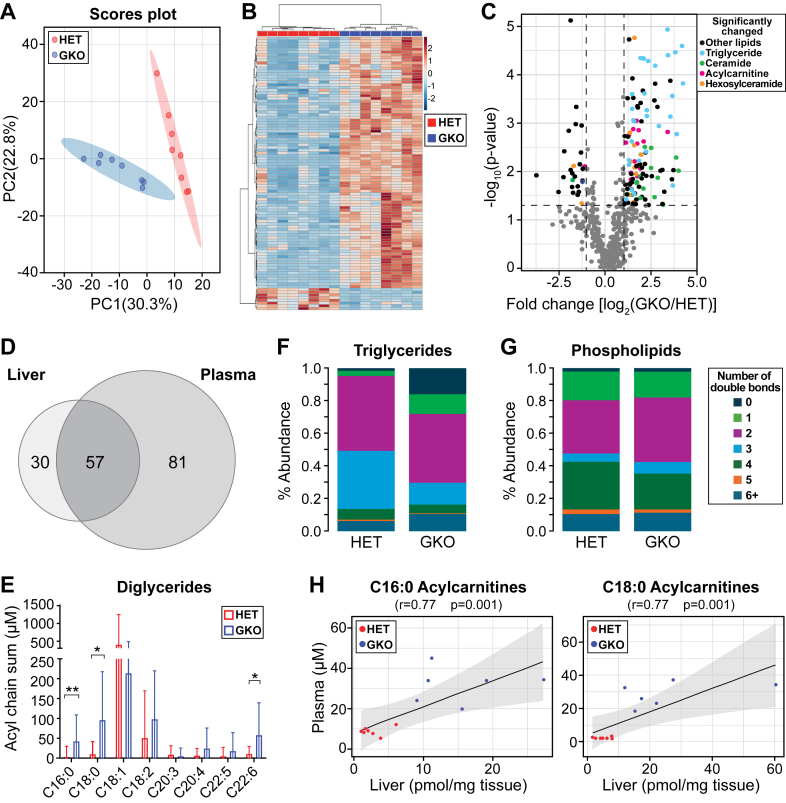
Global *Scd1* deficiency altered plasma lipids and increased saturated plasma lipids under an HCD. A: PCA of lipids between GKO and HET mice. B: Heatmap and cluster analysis of hepatic lipids. C: Volcano plot showing the significance and fold change between GKO and HET mice of liver lipids. The significance of the lipids was considered at an FDR-corrected *P* value of 0.1 and a fold change of 2. Dots on the right denote lipid increase in the GKO mice, whereas dots on the left denote lipid decrease in the GKO mice. D: Venn diagram displaying an overlap of lipid species increased in the GKO mice in the liver and plasma. E: Acyl chain sum of the diglycerides. Relative abundance for each degree of total desaturation (number of double bonds) in F: Triglycerides and G: Phospholipids. ∗*P <* 0.05 and ∗∗*P <* 0.01. Data are presented as mean + SD. H: Correlation analysis between liver and plasma for C16:0 acylcarnitines and C18:0 acylcarnitines. HET, heterozygote mice. N = 6–8 mice.

We extended our lipidomic analysis to investigate the impact of *Scd1* deficiency on plasma lipids. In agreement with findings in the liver, the PCA and heatmaps show that the plasma lipids clustered according to the genotypes (Fig. 2A, B). Notably, 167 lipids exhibited significant changes, with 139 lipids elevated and 28 reduced (Fig. 2C and supplemental Table S5). Strikingly, over 65% of the elevated lipids in the liver were also increased in plasma, encompassing acylcarnitines, phospholipids, cholesteryl esters, ceramides, and hexosylceramides (Fig. 2C-D; supplemental Table S5). Despite the general reduction in liver triglycerides, the primary fat storage form, there was a general increase in triglyceride species in the plasma of GKO mice (Fig. 2C). This finding suggests that under *Scd1* deficiency, the liver may shift from lipid storage to enhanced lipid export.

To determine whether changes in lipid saturation observed in the liver are also manifested in the plasma, we also analyzed the acyl chain distribution of diglycerides. This analysis revealed higher levels of C18:0 diglycerides in the plasma of GKO mice (Fig. 1E), indicative of a shift toward increased saturation. Moreover, examination of the double-bond distribution in triglycerides showed a marked increase in fully saturated triglycerides in GKO mice, reaching ∼16% compared with ∼1.5% in control mice (Fig. 1E). However, the levels of saturated phospholipids remained relatively unchanged between the two genotypes, at approximately 2% (Fig. 1F). The pronounced elevation of acylcarnitines in the plasma, including a remarkable 11-fold increase in saturated C18:0 acylcarnitines, prompted us to also investigate the relationship between hepatic and plasma saturated long-chain acylcarnitines. We identified a strong correlation between liver and plasma saturated C16:0 and C18:0 acylcarnitines (Fig. 2H). Collectively, these findings suggest that *Scd1* deficiency-induced alterations in liver lipid composition and saturation are reflected in circulating lipids.

### Human *SCD5* restores several lipidomic changes in the livers of global *Scd1-*deficient mice

**Fig. 3:**
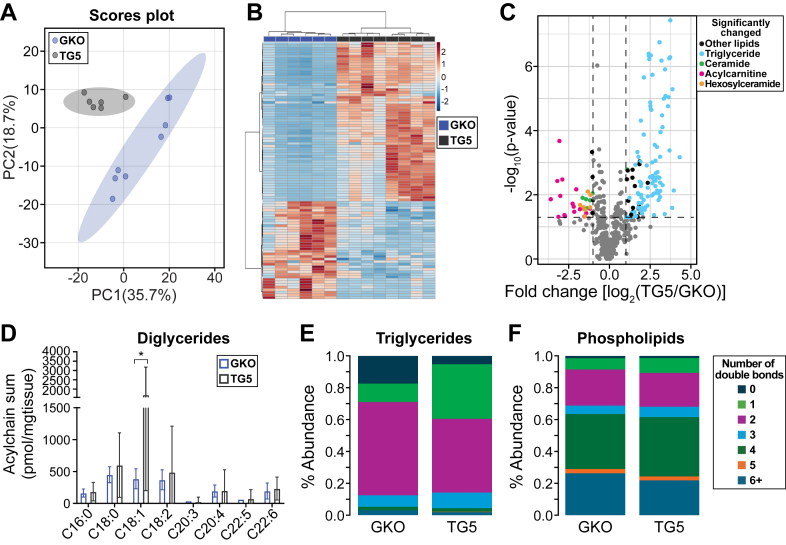
Expression of the human *SCD5* in the livers of GKO mice restores desaturation in the hepatic lipidome. A: PCA of lipids between 5TG and GKO. B: Heatmap and cluster analysis of hepatic lipids. C: Volcano plot showing the significance and fold change between 5TG and GKO mice of liver lipids. The significance of the lipids was considered at an FDR-corrected *P* value of 0.1 and a fold change of 2. Dots on the right denote lipid increase in the 5TG mice, whereas dots on the left denote lipid decrease in the 5TG mice. D: Acyl chain sum of the diglycerides. Relative abundance for each degree of total desaturation (number of double bonds) in E: Triglycerides and F: Phospholipids. ∗*P <* 0.05. 5TG, GKO mice with the expression of human *SCD5* in the liver. N = 6–8 mice.

We investigated the effect of replenishing endogenous oleate by the transgenic expression of the human *SCD5* isoform in the livers of GKO mice (24) on the hepatic lipid landscape of *Scd1*-deficient mice. SCD5 specifically catalyzes the Δ9-desaturation of stearate (C18:0) to oleate (C18:1n-9) by the introduction of a single double bond (24, 31). The PCA and heatmap showed that the hepatic lipids of these mice clustered according to their genotypes (Fig. 3A, B). We identified ∼500 lipids, of which 125 were significantly changed, with 89 increased and 36 decreased (Fig. 3C and supplemental Table S6). Notably, the introduction of *SCD5* restored triglyceride species, which were previously reduced in GKO mice (Fig. 3C and supplemental Fig. S2A). Furthermore, *SCD5* expression decreased the levels of acylcarnitines and several ceramide and hexosylceramide species in *Scd1*-deficient mice (Fig. 3C and supplemental Fig. S2). These findings demonstrate that human *SCD5* expression rescues the lipidomic perturbations induced by *Scd1* deficiency in the liver.

We then assessed the impact of expressing human *SCD5* on the saturation of hepatic lipids in GKO mice. Our results showed that *SCD5* expression led to a significant increase in the acyl chain of C18:1 in diglycerides (Fig. 3D), consistent with its role in oleate synthesis. Moreover, we observed a marked decrease in fully saturated triglycerides (∼5% in TG5 vs. ∼17% in GKO mice) and a concomitant increase in triglycerides with single double bonds (∼34% in TG5 vs. ∼12% in GKO mice) in TG5 mice. Additionally, a modest increase in phospholipids with single double bonds was observed in TG5 mice (∼19% TG5 vs. ∼16% in GKO mice). These findings collectively underscore the effect of *SCD5*, which synthesizes oleate, in regulating liver lipid desaturation and MUFA synthesis.

To examine the systemic impact of liver-specific transgenic human *SCD5* expression in GKO mice, we analyzed the plasma lipids of TG5 mice. Given the limited yield of significant lipids using FDR correction, we employed a non-FDR-corrected *P* value of 0.05, revealing 67 significant lipids, with 56 increased and 11 decreased (supplemental Fig. S3C and supplemental Table S7). We detected a striking overlap between the plasma and liver lipidomes, with over 78% of the elevated lipids in the plasma of TG5 mice also increased in the liver (supplemental Fig. S3D). In accordance with our hepatic findings, *SCD5* expression led to increased plasma triglycerides and decreased acylcarnitines (supplemental Fig. S3C and supplemental Table S7) as well as elevated levels of C18:1 in diglycerides (supplemental Fig. S3D). Furthermore, the plasma triglyceride profile of TG5 mice was characterized by a reduction in fully saturated lipids (∼6% in TG5 vs. ∼15% in GKO mice) and an increase in lipids with a single double bond (∼36% in TG5 vs. ∼12% in GKO mice) (supplemental Fig. S3E). Together, these findings demonstrate that *SCD5* expression impacts the hepatic lipidome and consequently circulating lipid profiles.

### Liver-specific *Scd1* deficiency increases hepatic and circulating acylcarnitines

**Fig. 4:**
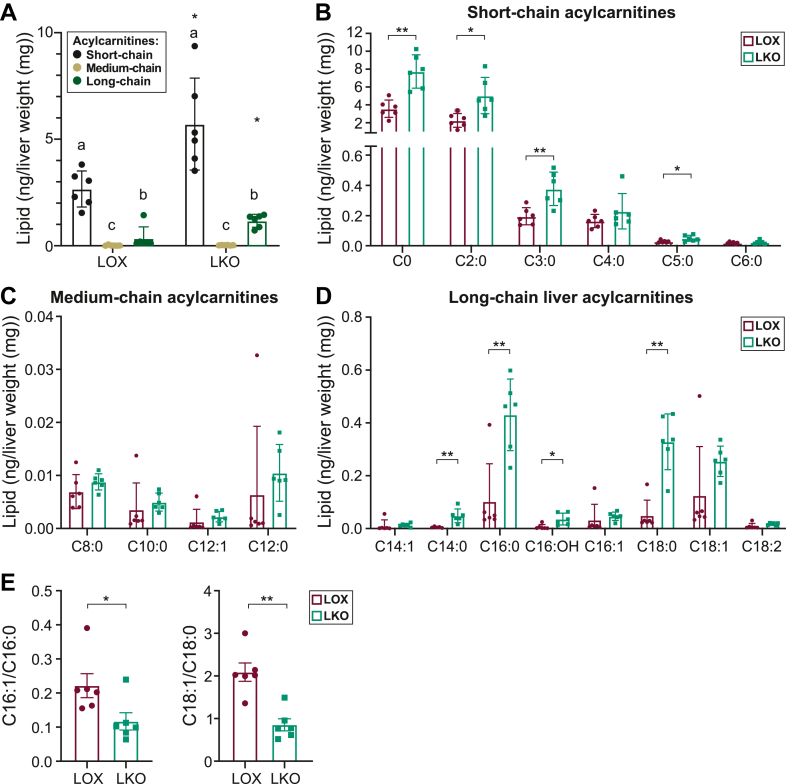
Liver-specific *Scd1* deficiency increases hepatic acylcarnitines. A: Pooled liver short-, medium-, and long-chain acylcarnitines. B: Liver carnitine (C0) and short-chain acylcarnitines. C: Liver medium-chain acylcarnitines. D: Liver long-chain acylcarnitines. E: C16:1/C16:0 and C18:1/C18:0 desaturation indices. ∗*P <* 0.05, ∗∗*P <* 0.01, and *∗∗∗P <* 0.001. Data are presented as mean ± SD. Means not connected by the same letter within a genotype were significantly different. ∗Denotes differences between genotypes within the same chain length. N = 6 mice. LOX, Lox control mice.

To validate further the increase in liver and plasma acylcarnitines, we utilized targeted lipidomics in the liver of the LKO mice fed an HCD diet. Investigating acylcarnitines is crucial as they play a key role in metabolism, with implications both in health and disease (32). When pooled together, our findings in the liver showed that the short-chain acylcarnitines were the most abundant, followed by the long-chain acylcarnitines, and the medium-chain acylcarnitines were the least abundant among the identified acylcarnitines in both LKO and LOX mice groups (Fig. 4A). The LKO mice exhibited elevated liver carnitine, short-chain acylcarnitines (C2:0, C3:0, and C5:0) (Fig. 4B), and long-chain acylcarnitines (C14:0, C16:0, and C18:0) (Fig. 4C) with no discernible changes in the medium-chain acylcarnitines (Fig. 4C). The detected increase in saturated acylcarnitines in the liver lipids of LKO mice (Fig. 4D) was consistent with their lower 16:1/16:0 and 18:1/18:0 desaturation indices (Fig. 4E).

**Fig. 5:**
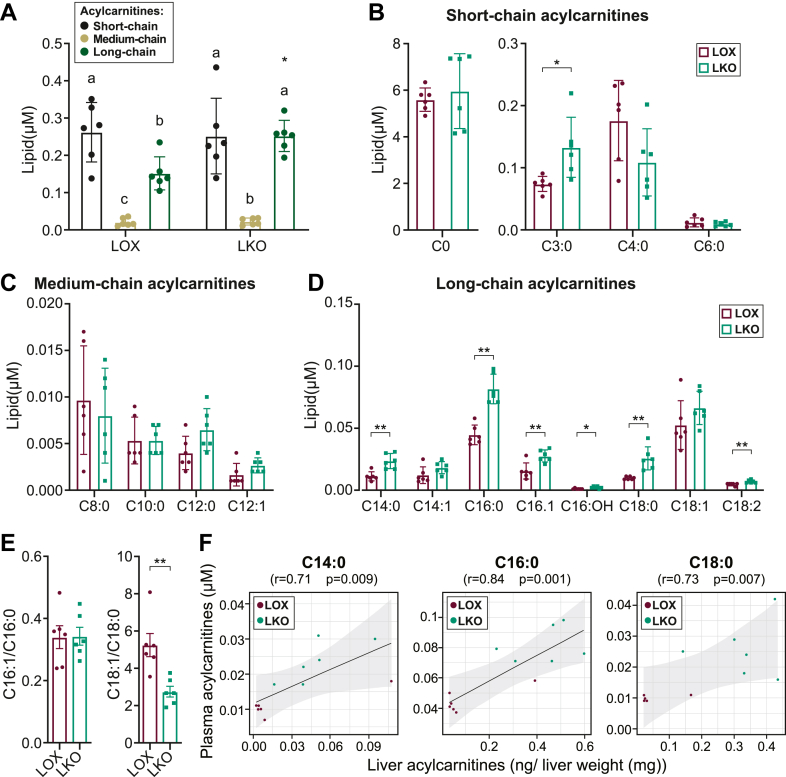
Liver-specific *Scd1* deficiency increases circulating acylcarnitines. A: Pooled plasma short, medium, and long chain acylcarnitines. B: Plasma carnitine and short-chain acylcarnitines, C: Plasma medium-chain acylcarnitines, and D: Plasma long-chain acylcarnitines. E: C16:1/C16:0 and C18:1/C18:0 desaturation indices. F: Correlation analysis between liver and plasma for C14:0 acylcarnitines, C16:0 acylcarnitines, and C18:0 acylcarnitines. ∗*P <* 0.05, ∗∗*P <* 0.01, *∗∗∗P <* 0.001. Data are presented as Mean ± SD. Means not connected by the same letter within a genotype were significantly different. ∗Denotes differences between genotypes within the same chain length. N = 6 mice. LOX, Lox control mice.

To determine whether the hepatic findings were mirrored in the circulating lipids, we performed the plasma lipidomic analysis. When pooled together, the short-chain acylcarnitines were the most abundant, followed by long-chain acylcarnitines and medium-chain acylcarnitines being the least abundant in the LOX group (Fig. 5A). We also detected elevated long-chain acylcarnitines (C14:0, C16:0, and C18:0) in the LKO mice (Fig. 5D) accompanied by a lower C18:1/18:0 desaturation index (Fig. 5E). Further correlation analysis revealed a strong correlation between liver long-chain saturated acylcarnitines and plasma long-chain saturated acylcarnitines (Fig. 5F). Our findings indicate that *Scd1* deficiency increased hepatic long-chain acylcarnitines, which likely led to the increase in circulating long-chain acylcarnitines.

### *Scd1* deficiency downregulates hepatic gene expression related to acylcarnitine metabolism, restored by human *SCD5* expression

**Fig. 6:**
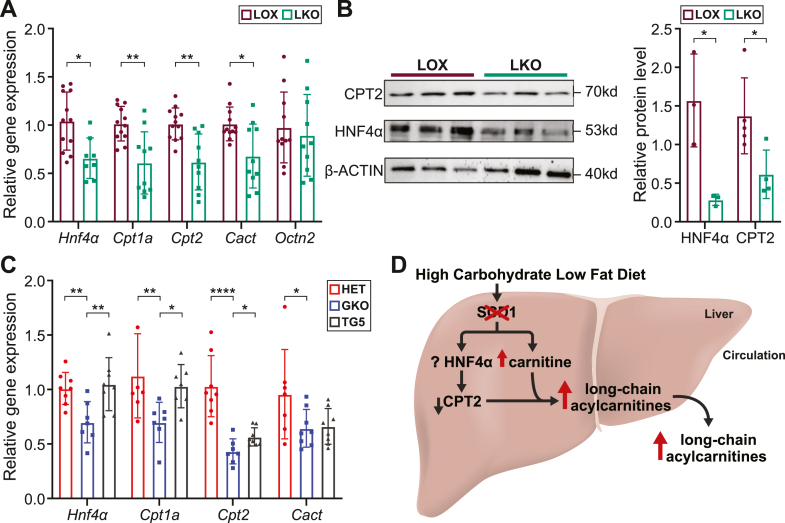
Liver-specific *Scd1* deficiency alters the expression of hepatic genes related to acylcarnitine metabolism. A: Relative expression of hepatic genes related to acylcarnitine metabolism in LKO and LOX mice N = 9–12 mice. B: Western blot and associated densitometric quantification of liver CPT2, HNF4α, and β-ACTIN. N = 3–5 mice. C: Relative expression of hepatic genes related to acylcarnitine metabolism in HET, GKO, and TG5 mice. N = 6–8 mice. D: Summary: By elevating liver carnitine and lowering liver Cpt2, hepatic *Scd1* deficiency under an HCD leads to increased long-chain acylcarnitines in the liver and the circulation. *Cact*, carnitine/acylcarnitine translocase (solute carrier family 25 member 20); *Cpt1a*, carnitine palmitoyltransferase 1A; *Cpt2*, carnitine palmitoyltransferase 2; *Hnf4α*, hepatocyte nuclear factor 4 alpha; *Octn2*, solute carrier family 22 member 5. ∗*P <* 0.05, ∗∗*P <* 0.01, *∗∗∗P <* 0.001, and *∗∗∗∗P <* 0.0001. Data are presented as mean + SD. HET, heterozygote mice; LOX, Lox control mice; 5TG, GKO mice with the expression of human *SCD5* in the liver.

After detecting an increase in both hepatic and circulating acylcarnitines under *Scd1* deficiency, we assessed the expression of genes related to acylcarnitine metabolism. Interestingly, ChIP-Seq analysis in the mouse liver revealed HNF4α binding around the *Scd1* gene loci, particularly in the upstream region (supplemental Fig. S4A). Additionally, ChIP-Seq confirmed robust HNF4α binding to established targets, including *Cpt1α* and *Cact* (supplemental Fig. S4B-D). Further analysis revealed that LKO mice exhibited a decrease in the hepatic expression of *Hnf4α* and its target genes, carnitine palmitoyl transferase 1A (*Cpt1a*), carnitine palmitoyl transferase 2 (*Cpt2*), and carnitine-acylcarnitine translocase (*Cact*), in the LKO mice (Fig. 6A). Furthermore, Western blot analysis revealed a decrease in the protein levels of HNF4α and CPT2 (Fig. 6B). Deficiency of CPT2 and CACT has been associated with an increase in long-chain acylcarnitines (32, 33). Although correlative, the decrease in the expression of markers related to acylcarnitine metabolism could have impacted acylcarnitine levels in *Scd1*-deficient mice.

Since our lipidomics analysis revealed a decrease in acylcarnitine levels following the restoration of endogenous oleate synthesis via the introduction of the human *SCD5* isoform in the livers of *Scd1*-deficient mice, we sought to investigate the expression of genes related to acylcarnitine metabolism in the TG5 mice. Intriguingly, reintroduction of human *SCD5* restored the expression of key markers related to acylcarnitine metabolism, including *Hnf4α*, *Cpt1a*, and *Cpt2* (Fig. 6C). These findings suggest that the expression of human *SCD5*, which synthesizes oleate, plays a significant role in modulating acylcarnitine levels under *Scd1* deficiency, revealing a key role of SCD1 and oleate in regulating acylcarnitine metabolism.

## Discussion

SCD1 is integral to lipogenesis, catalyzing the conversion of saturated fatty acids to MUFAs. Our lipidomic analysis revealed that *Scd1* deficiency profoundly alters the hepatic lipidome, leading to a marked increase in saturated lipids. Furthermore, we detected extensive alterations of plasma lipids, including elevated saturated lipids, suggesting that hepatic lipid alterations under *Scd1* deficiency have systemic effects. Intriguingly, our studies showed that *Scd1* deficiency led to an elevation of hepatic and plasma acylcarnitines. Notably, the reintroduction of endogenous oleate synthesis via transgenic expression of human *SCD5* in the livers of *Scd1*-deficient mice restored lipid desaturation and mitigated acylcarnitine accumulation. Overall, this work broadens our understanding of the metabolic role of SCD1, uncovering a previously unrecognized role of SCD1 in regulating acylcarnitine metabolism.

Our previous studies demonstrated that *Scd1* deficiency not only lowers body weight but also alleviates hepatic steatosis through the downregulation of hepatic de novo lipogenesis (1, 23). Findings from other studies also showed that transcriptionally repressing mammalian desaturases, including SCD, Δ5D, and Δ6D desaturase, through omega-3 long-chain polyunsaturated fatty acid supplementation, found in extra virgin oil, also protects against fatty liver under a lipogenic diet (34, 35, 36). The protective effect against fatty liver could partly be due to a significant decrease in liver total triglycerides, key components of lipid storage, as demonstrated by our findings after *Scd1* ablation (1). Lipidomic analysis in this study further revealed that loss of *Scd1* reduced several triglyceride species, potentially accounting for the overall reduction in triglycerides. Beyond triglycerides, our study shows that *Scd1* deficiency exerts a broader metabolic impact, modulating several lipid species, such as diglycerides, ceramides, and acylcarnitines, all of which are known to affect insulin signaling (32, 37, 38). Notably, several alterations in hepatic lipids induced by SCD1 were mirrored in circulating lipids, rendering a plausible explanation underlying the extrahepatic effects of liver-specific *Scd1* deficiency (1, 22).

Expressing the human *SCD5* in the livers of *Scd1* GKO mice reversed several metabolic effects of *Scd1* ablation, such as protection against obesity, hyperglycemia, and hepatic steatosis (24). SCD5 encodes a functional delta 9 desaturase and specifically catalyzes the conversion of stearate (C18:0) to oleate (C18:1n-9), mainly in the brain and pancreas (24, 31). Moreover, our previous findings employing GC showed that transgenic *SCD5* in the mouse liver of *Scd1*-deficient mice increased 18:0 desaturation, leading to elevated hepatic 18:1n-9 (24). Lipidomic analysis in the current study further revealed that endogenous oleate replenishment by SCD5 also restored triglycerides in addition to several other lipids that had not been previously implicated in SCD1-mediated metabolic regulation, including ceramides, hexosylceramides, and acylcarnitines, which were intriguingly reflected in the circulation. These findings collectively highlight the pivotal role of SCD1 and its product, oleate, in modulating lipid metabolism.

Our lipidomics analysis also revealed a profound elevation of long-chain acylcarnitines, key players in lipid metabolism, both in the liver and plasma of the global *Scd1*-deficient mice. We then employed targeted lipidomics to interrogate the effect of *Scd1* deficiency in LKO mice to rule out the contribution of other tissues, uncovering a significant elevation in liver L-carnitine content consistent with our metabolomic analysis (39). Notably, L-carnitine, a Food and Drug Administration-approved compound for treating carnitine deficiency (32), is a required substrate for the carnitine acyltransferase system, which catalyzes the esterification of fatty acids to form acylcarnitines. We detected a concomitant increase in the levels of long-chain acylcarnitines, including the saturated C16:0 and C18:0 acylcarnitines, both in the LKO livers and plasma, highlighting the liver’s critical role in shaping the circulating acylcarnitine pool. Since the reversible acyltransferase reaction depends on substrates like carnitine (40), the increased availability of carnitine in the livers of LKO mice (39) likely played a role in the increase in acylcarnitines in the livers and plasma of the *Scd1*-deficient mice.

To gain an insight into how *Scd1* deficiency elevated acylcarnitines, we examined the expression of genes and proteins orchestrating acylcarnitine metabolism. We detected a decrease in the expression of *Hnf4α* and its target genes *Cpt1a*, *Cpt2*, and *Cact* (41, 42, 43) in the livers of *Scd1*-deficient mice. CPT1 synthesizes long-chain acylcarnitines by conjugating carnitine to long-chain fatty acids, which are transported across the inner mitochondrial membrane by the CACT transporter, followed by removal of carnitine by CPT2 in the mitochondrial matrix for subsequent oxidation (42). CPT2 and CACT deficiency results in an accumulation of long-chain acylcarnitines (33, 44, 45). Interestingly, SCD5-mediated endogenous restoration of oleate restored the expression of acylcarnitine-related genes, such as *Hnf4α*, *Cpt1*, and *Cpt2*. The concurrent increase in liver carnitine availability, coupled with reduced expression of CPT2, likely facilitated the marked accumulation of long-chain acylcarnitines in the livers of *Scd1*-deficient mice, which were then released into the plasma (Fig. 6C).

Acylcarnitines are elevated in metabolic stresses, such as fasting, exercise, cold exposure, insulin resistance, diabetes, and inflammation (32, 42). *Scd1*-deficient mice present with not only improved insulin sensitivity but also increased inflammation (1, 23, 46). The marked elevation of saturated long-chain acylcarnitines in *Scd1*-deficient mice is associated with proinflammatory signals (47, 48), thus implying that their accumulation likely contributes to the hepatic inflammatory phenotype under *Scd1* deficiency. On the other hand, *Scd1* deficiency boosts fatty acid oxidation in white adipose tissue and augments basal thermogenesis by upregulating the expression of uncoupling proteins in the brown adipose tissue (49, 50). It is plausible that hepatic-generated long-chain acylcarnitines under *Scd1* deficiency could be circulated to extrahepatic tissues such as white adipose tissue to boost lipid oxidation and brown adipose tissue to serve as substrates for thermogenesis as observed under cold exposure (42, 50). Our findings thus provide insights into the role of SCD1 in physiological and pathological contexts, which are likely useful in the design and testing of therapies that target SCD1.

Our investigations sought to deepen our comprehension of the effect of SCD1 on lipid metabolism. Together, our findings demonstrate that *Scd1* deficiency dramatically alters lipid abundance and saturation in the liver, effects mirrored in the circulating lipid profile. We also demonstrated that *Scd1* deficiency enhances hepatic and circulating acylcarnitines, underscoring the significant role of SCD1 in acylcarnitine metabolism. Notably, the endogenous replenishment of oleate through the transgenic expression of human *SCD5* in the livers of *Scd1*-deficient mice restored lipid desaturation and acylcarnitine levels. These studies broaden our understanding of the role of SCD1 and its product, oleate, in regulating lipid metabolism.

## Data availability

All data are contained within the article and the accompanying supplemental data. Lipidomics raw data were uploaded to MassIVE (#MSV000096321).

## Supplemental data

This article contains supplemental data.

## Conflict of interest

The author declares that they have no conflicts of interest with the contents of this article.

## References

[1] Miyazaki M., Flowers M.T., Sampath H., Chu K., Otzelberger C., Liu X. (2007). Hepatic stearoyl-CoA desaturase-1 deficiency protects mice from carbohydrate-induced adiposity and hepatic steatosis. Cell Metab..

[2] ALJohani A.M., Syed D.N., Ntambi J.M. (2017). Insights into stearoyl-CoA desaturase-1 regulation of systemic metabolism. Trends Endocrinol. Metab..

[3] Cases S., Smith S.J., Zheng Y.-W., Myers H.M., Lear S.R., Sande E. (1998). Identification of a gene encoding an acyl CoA:diacylglycerol acyltransferase, a key enzyme in triacylglycerol synthesis. Proc. Natl. Acad. Sci. U. S. A..

[4] Miyazaki M., Ntambi J.M. (2003). Role of stearoyl-coenzyme A desaturase in lipid metabolism. Prostaglandins Leukot. Essent. Fatty Acids.

[5] Castro L.F.C., Wilson J.M., Gonçalves O., Galante-Oliveira S., Rocha E., Cunha I. (2011). The evolutionary history of the stearoyl-CoA desaturase gene family in vertebrates. BMC Evol. Biol..

[6] Sun Q., Xing X., Wang H., Wan K., Fan R., Liu C. (2024). SCD1 is the critical signaling hub to mediate metabolic diseases: mechanism and the development of its inhibitors. Biomed. Pharmacother..

[7] Sampath H., Ntambi J.M. (2005). Polyunsaturated fatty acid regulation of genes of lipid metabolism. Annu. Rev. Nutr..

[8] Jones B.H., Maher M.A., Banz W.J., Zemel M.B., Whelan J., Smith P.J. (1996). Adipose tissue stearoyl-CoA desaturase mRNA is increased by obesity and decreased by polyunsaturated fatty acids. Am. J. Physiol. Endocrinol. Metab..

[9] Biddinger S.B., Miyazaki M., Boucher J., Ntambi J.M., Kahn C.R. (2006). Leptin suppresses stearoyl-CoA desaturase 1 by mechanisms independent of insulin and sterol regulatory element–binding protein-1c. Diabetes.

[10] Ntambi J.M. (1999). Regulation of stearoyl-CoA desaturase by polyunsaturated fatty acids and cholesterol. J. Lipid Res..

[11] Hulver M.W., Berggren J.R., Carper M.J., Miyazaki M., Ntambi J.M., Hoffman E.P. (2005). Elevated stearoyl-CoA desaturase-1 expression in skeletal muscle contributes to abnormal fatty acid partitioning in obese humans. Cell Metab..

[12] Suppli M.P., Rigbolt K.T.G., Veidal S.S., Heebøll S., Eriksen P.L., Demant M. (2019). Hepatic transcriptome signatures in patients with varying degrees of nonalcoholic fatty liver disease compared with healthy normal-weight individuals. Am. J. Physiol. Gastrointestinal Liver Physiol..

[13] Ran H., Zhu Y., Deng R., Zhang Q., Liu X., Feng M. (2018). Stearoyl-CoA desaturase-1 promotes colorectal cancer metastasis in response to glucose by suppressing PTEN. J. Exp. Clin. Cancer Res..

[14] García-Serrano S., Moreno-Santos I., Garrido-Sánchez L., Gutierrez-Repiso C., García-Almeida J.M., García-Arnés J. (2011). Stearoyl-CoA desaturase-1 is associated with insulin resistance in morbidly obese subjects. Mol. Med..

[15] Younossi Z.M., Golabi P., Paik J.M., Henry A., Van Dongen C., Henry L. (2023). The global epidemiology of nonalcoholic fatty liver disease (NAFLD) and nonalcoholic steatohepatitis (NASH): a systematic review. Hepatology.

[16] Warensjö E., Ingelsson E., Lundmark P., Lannfelt L., Syvänen A.-C., Vessby B. (2007). Polymorphisms in the SCD1 gene: associations with body fat distribution and insulin sensitivity. Obesity (Silver Spring).

[17] Liew C.F., Groves C.J., Wiltshire S., Zeggini E., Frayling T.M., Owen K.R. (2004). Analysis of the contribution to type 2 diabetes susceptibility of sequence variation in the gene encoding stearoyl-CoA desaturase, a key regulator of lipid and carbohydrate metabolism. Diabetologia.

[18] Oballa R.M., Belair L., Black W.C., Bleasby K., Chan C.C., Desroches C. (2011). Development of a liver-targeted stearoyl-CoA desaturase (SCD) inhibitor (MK-8245) to establish a therapeutic window for the treatment of diabetes and dyslipidemia. J. Med. Chem..

[19] Ratziu V., de Guevara L., Safadi R., Poordad F., Fuster F., Flores-Figueroa J. (2021). Aramchol in patients with nonalcoholic steatohepatitis: a randomized, double-blind, placebo-controlled phase 2b trial. Nat. Med..

[20] Sen U., Coleman C., Sen T. (2023). Stearoyl coenzyme A desaturase-1: multitasker in cancer, metabolism, and ferroptosis. Trends Cancer.

[21] Ntambi J.M., Miyazaki M., Stoehr J.P., Lan H., Kendziorski C.M., Yandell B.S. (2002). Loss of stearoyl–CoA desaturase-1 function protects mice against adiposity. Proc. Natl. Acad. Sci. U. S. A..

[22] Aljohani A., Khan M.I., Bonneville A., Guo C., Jeffery J., O’Neill L. (2019). Hepatic stearoyl CoA desaturase 1 deficiency increases glucose uptake in adipose tissue partially through the PGC-1α–FGF21 axis in mice. J. Biol. Chem..

[23] Flowers M.T., Keller M.P., Choi Y., Lan H., Kendziorski C., Ntambi J.M. (2008). Liver gene expression analysis reveals endoplasmic reticulum stress and metabolic dysfunction in SCD1-deficient mice fed a very low-fat diet. Physiol. Genomics.

[24] Burhans M.S., Flowers M.T., Harrington K.R., Bond L.M., Guo C.-A., Anderson R.M. (2015). Hepatic oleate regulates adipose tissue lipogenesis and fatty acid oxidation. J. Lipid Res..

[25] Livak K.J., Schmittgen T.D. (2001). Analysis of relative gene expression data using real-time quantitative PCR and the 2−ΔΔCT method. Methods..

[26] Huynh K., Barlow C.K., Jayawardana K.S., Weir J.M., Mellett N.A., Cinel M. (2019). High-throughput plasma lipidomics: detailed mapping of the associations with cardiometabolic risk factors. Cell Chem. Biol..

[27] Von Bank H., Geoghegan G., Jain R., Kotulkar M., Hurtado-Thiele M., Gonzalez P. (2023). HNF4α regulates acyl chain remodeling and ether lipid accumulation in hepatic steatosis. bioRxiv.

[28] Pang Z., Chong J., Zhou G., de Lima Morais D.A., Chang L., Barrette M. (2021). MetaboAnalyst 5.0: narrowing the gap between raw spectra and functional insights. Nucleic Acids Res..

[29] Yu D., Shu X.-O., Li H., Xiang Y.-B., Yang G., Gao Y.-T. (2013). Dietary carbohydrates, refined grains, glycemic load, and risk of coronary heart disease in Chinese adults. Am. J. Epidemiol..

[30] O’Hearn M., Lara-Castor L., Cudhea F., Miller V., Reedy J., Shi P. (2023). Incident type 2 diabetes attributable to suboptimal diet in 184 countries. Nat. Med..

[31] Wang J., Yu L., Schmidt R.E., Su C., Huang X., Gould K. (2005). Characterization of HSCD5, a novel human stearoyl-CoA desaturase unique to primates. Biochem. Biophys. Res. Commun..

[32] Dambrova M., Makrecka-Kuka M., Kuka J., Vilskersts R., Nordberg D., Attwood M.M. (2022). Acylcarnitines: nomenclature, biomarkers, therapeutic potential, drug targets, and clinical trials. Pharmacol. Rev..

[33] Iacobazzi V., Pasquali M., Singh R., Matern D., Rinaldo P., di San Filippo C.A. (2004). Response to therapy in carnitine/acylcarnitine translocase (CACT) deficiency due to a novel missense mutation. Am. J. Med. Genet. A..

[34] Rincón-Cervera M.A., Valenzuela R., Hernandez-Rodas M.C., Marambio M., Espinosa A., Mayer S. (2016). Supplementation with antioxidant-rich extra virgin olive oil prevents hepatic oxidative stress and reduction of desaturation capacity in mice fed a high-fat diet: effects on fatty acid composition in liver and extrahepatic tissues. Nutrition.

[35] Videla L.A., Hernandez-Rodas M.C., Metherel A.H., Valenzuela R. (2022). Influence of the nutritional status and oxidative stress in the desaturation and elongation of n-3 and n-6 polyunsaturated fatty acids: impact on non-alcoholic fatty liver disease. Prostaglandins Leukot. Essent. Fatty Acids.

[36] Nakamura M.T., Nara T.Y. (2002). Gene regulation of mammalian desaturases. Biochem. Soc. Trans..

[37] Chaurasia B., Summers S.A. (2015). Ceramides – lipotoxic inducers of metabolic disorders. Trends Endocrinol. Metab..

[38] Perry R.J., Samuel V.T., Petersen K.F., Shulman G.I. (2014). The role of hepatic lipids in hepatic insulin resistance and type 2 diabetes. Nature.

[39] Ntambi J.M., Liu X., Burhans M.S., ALjohani A., Selen E.S., Kalyesubula M. (2023). Hepatic oleate regulates one-carbon metabolism during high carbohydrate feeding. Biochem. Biophys. Res. Commun..

[40] Houten S.M., Wanders R.J.A., Ranea-Robles P. (2020). Metabolic interactions between peroxisomes and mitochondria with a special focus on acylcarnitine metabolism. Biochim. Biophys. Acta Mol. Basis Dis..

[41] Hayhurst G.P., Lee Y.-H., Lambert G., Ward J.M., Gonzalez F.J. (2001). Hepatocyte nuclear factor 4α (nuclear receptor 2A1) is essential for maintenance of hepatic gene expression and lipid homeostasis. Mol. Cell Biol..

[42] Simcox J., Geoghegan G., Maschek J.A., Bensard C.L., Pasquali M., Miao R. (2017). Global analysis of plasma lipids identifies liver-derived acylcarnitines as a fuel source for Brown fat thermogenesis. Cell Metab..

[43] Martinez-Jimenez C.P., Kyrmizi I., Cardot P., Gonzalez F.J., Talianidis I. (2010). Hepatocyte nuclear factor 4α coordinates a transcription factor network regulating hepatic fatty acid metabolism. Mol. Cell Biol..

[44] Pereyra A.S., Rajan A., Ferreira C.R., Ellis J.M. (2020). Loss of muscle carnitine palmitoyltransferase 2 prevents diet-induced obesity and insulin resistance despite long-chain acylcarnitine accumulation. Cell Rep..

[45] Lee J., Choi J., Scafidi S., Wolfgang M.J. (2016). Hepatic fatty acid oxidation restrains systemic catabolism during starvation. Cell Rep..

[46] Liu X., Burhans M.S., Flowers M.T., Ntambi J.M. (2016). Hepatic oleate regulates liver stress response partially through PGC-1α during high-carbohydrate feeding. J. Hepatol..

[47] McCoin C.S., Knotts T.A., Ono-Moore K.D., Oort P.J., Adams S.H. (2015). Long-chain acylcarnitines activate cell stress and myokine release in C2C12 myotubes: calcium-dependent and -independent effects. Am. J. Physiol. Endocrinol. Metab..

[48] Rutkowsky J.M., Knotts T.A., Ono-Moore K.D., McCoin C.S., Huang S., Schneider D. (2014). Acylcarnitines activate proinflammatory signaling pathways. Am. J. Physiol. Endocrinol. Metab..

[49] Lee S.-H., Dobrzyn A., Dobrzyn P., Rahman S.M., Miyazaki M., Ntambi J.M. (2004). Lack of stearoyl-CoA desaturase 1 upregulates basal thermogenesis but causes hypothermia in a cold environment. J. Lipid Res..

[50] Miyazaki M., Dobrzyn A., Sampath H., Lee S.-H., Man W.C., Chu K. (2004). Reduced adiposity and liver steatosis by stearoyl-CoA desaturase deficiency are independent of peroxisome proliferator-activated receptor-α. J. Biol. Chem..

