# Supplementary figures and images for: Stearoyl-CoA desaturase 1 deficiency drives saturated lipid accumulation and increases liver and plasma acylcarnitines
Source: J Lipid Res. 2025 May 9;66(6):100824. doi: 10.1016/j.jlr.2025.100824 (PMC12173144; doi:10.1016/j.jlr.2025.100824)

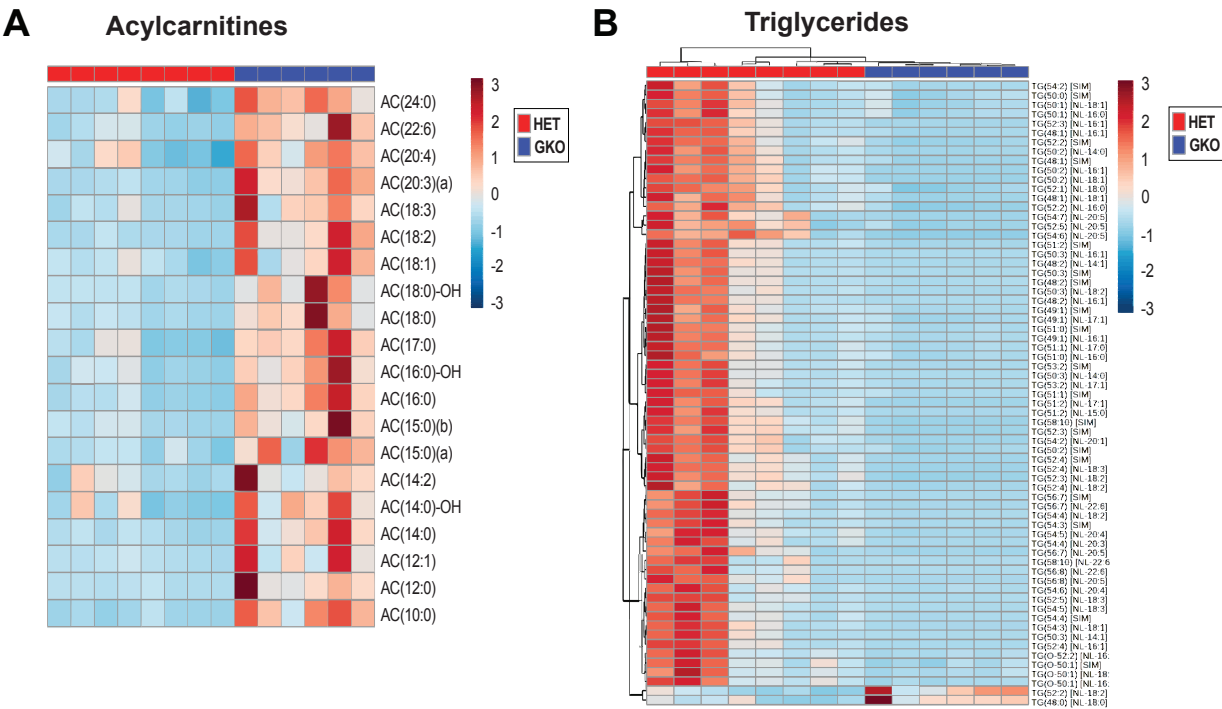

Supplement: Supplementary Figure 1 [file mmc1.pdf]

Supplementary Figure 4

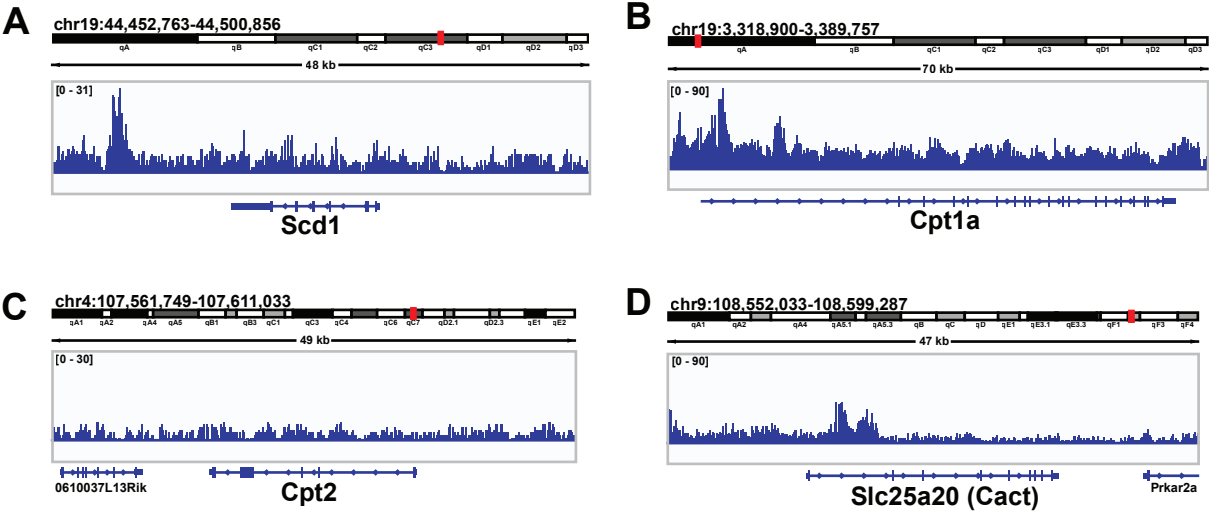

Supplementary Figure 4: HNF4α occupancy tracks on Scd1, Cpt1a, Cpt2, and Slc25a20

Supplement: Supplementary Figure 4 [file mmc4.pdf]
