# Supplementary material for: Stearoyl-CoA desaturase 1 deficiency drives saturated lipid accumulation and increases liver and plasma acylcarnitines
Source: J Lipid Res. 2025 May 9;66(6):100824. doi: 10.1016/j.jlr.2025.100824 (PMC12173144; doi:10.1016/j.jlr.2025.100824)
Supplement: Supplementary Figure 2 [file mmc2.pdf]

Supplementary Figure 2

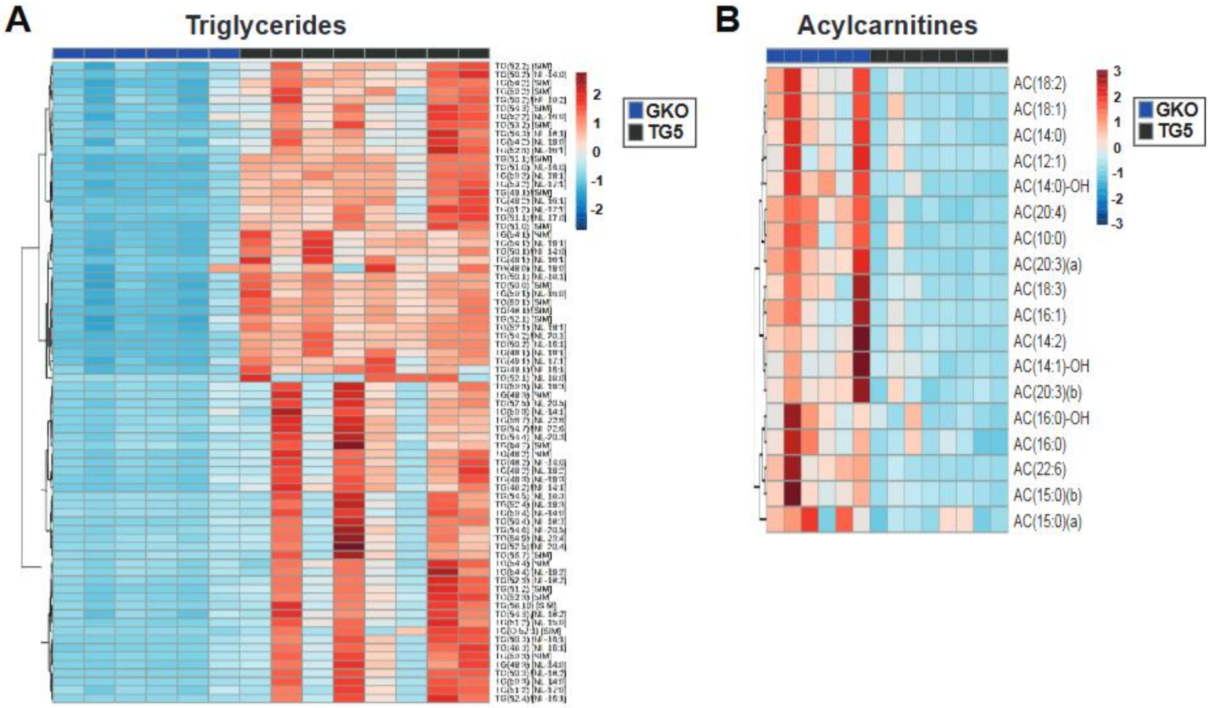

Supplementary Figure 2: Heat map of the significantly altered A: acylcarnitines and B: triglycerides

GKO: Global *Scd1* deficient mice, 5TG: GKO mice with the expression of human *SCD5* in the liver.
