# Supplementary material for: Stearoyl-CoA desaturase 1 deficiency drives saturated lipid accumulation and increases liver and plasma acylcarnitines
Source: J Lipid Res. 2025 May 9;66(6):100824. doi: 10.1016/j.jlr.2025.100824 (PMC12173144; doi:10.1016/j.jlr.2025.100824)
Supplement: Supplementary Figure 3 [file mmc3.pdf]

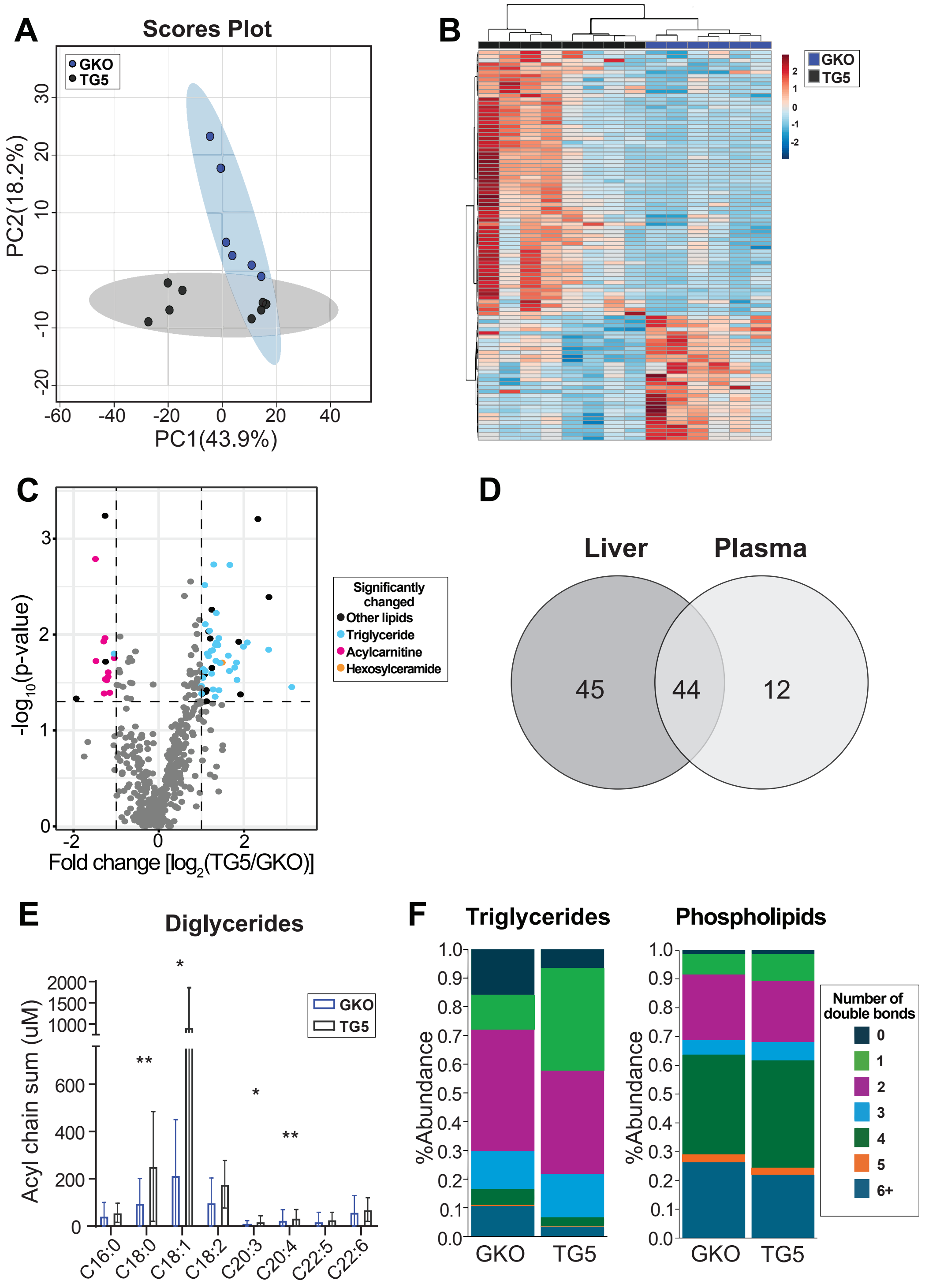

Supplementary Figure 3: The effect of expressing the human *SCD5* isoform in the GKO mice on the circulating plasma lipids. A: Principal component analysis of lipids between 5TG and GKO. B: Heat map and cluster analysis of hepatic lipids. C: Volcano plot showing the significance and fold change between 5TG and GKO mice of liver lipids. The significance of the lipids was considered at a *P* value of 0.05 and a fold change of 2. Dots on the right denote lipid increase in the 5TG mice, while dots on the left denote lipid decrease in the 5TG mice. D: Venn diagram displaying an overlap of lipid species increased in the TG5 mice in the liver and plasma. E: Acyl chain sum of the diglycerides. Relative abundance for each degree of total desaturation (number of double bonds) in F: Triglycerides and G: Phospholipids. *P< 0.05, **P< 0.01. GKO: Global *Scd1* deficient mice, TG5: GKO mice with the expression of human *SCD5* in the liver. N = 6-8 mice.
