# Supplementary material for: Stearoyl-CoA desaturase 1 deficiency drives saturated lipid accumulation and increases liver and plasma acylcarnitines
Source: J Lipid Res. 2025 May 9;66(6):100824. doi: 10.1016/j.jlr.2025.100824 (PMC12173144; doi:10.1016/j.jlr.2025.100824)
Supplement: Supplementary Table 1 [file mmc5.docx]

Supplementary Table 1. List of real-time PCR Primers listed 5’ to 3’

| Gene | Forward | Reverse |
| --- | --- | --- |
| *Cpt1a* | TTGGAAGTCTCCCTCCTTCA | GCCCATGTTGTACAGCTTCC |
| *Cact* | GGTGGCTGTCCAGACAAACT | TCCGTTTAAGAACCTCCTGG |
| *Cpt2* | TCTTCCTGAACTGGCTGTCA | GTACCCACCATGCACTACCA |
| *Octn2* | AAGACCTGCAGGAAGCTGAA | TCCTTGTTTTTCGTGGGTGT |
