# Supplementary material for: Stearoyl-CoA desaturase 1 deficiency drives saturated lipid accumulation and increases liver and plasma acylcarnitines
Source: J Lipid Res. 2025 May 9;66(6):100824. doi: 10.1016/j.jlr.2025.100824 (PMC12173144; doi:10.1016/j.jlr.2025.100824)
Supplement: Supplementary Table 2 [file mmc6.docx]

Supplementary Table 2. Parameters for liquid chromatography gradient for acylcarnitine analysis.

|  | **Time (min)** | **Mobile A (%)** | **Mobile B (%)** | **Flow rate (mL/min)** |
| --- | --- | --- | --- | --- |
| **1** | 0.80 min | 70.00 % | 30.00 % | 0.500 |
| **2** | 2.20 min | 40.00 % | 60.00 % | 0.500 |
| **3** | 8.00 min | 20.00 % | 80.00 % | 0.500 |
| **4** | 8.14 min | 1.00 % | 99.00 % | 0.500 |
| **5** | 9.60 min | 1.00 % | 99.00 % | 0.500 |
| **6** | 10.00 min | 85.00 % | 15.00 % | 0.500 |
