# Supplementary material for: Stearoyl-CoA desaturase 1 deficiency drives saturated lipid accumulation and increases liver and plasma acylcarnitines
Source: J Lipid Res. 2025 May 9;66(6):100824. doi: 10.1016/j.jlr.2025.100824 (PMC12173144; doi:10.1016/j.jlr.2025.100824)
Supplement: Supplementary Table 3 [file mmc7.docx]

Supplementary Table 3. Parameters for dMRM analysis of acylcarnitines.

| **Cpd Name** | **ISTD?** | **Prec Ion** | **Prod Ion** | **Frag (V)** | **CE (V)** | **Cell Acc (V)** | **Ret Time (min)** | **Ret Window** | **Polarity** |
| --- | --- | --- | --- | --- | --- | --- | --- | --- | --- |
| C0 | No | 162 | 85.1 | 166 | 23 | 5 | 0.58 | 1.2 | Positive |
| C10 | No | 316.3 | 85.1 | 166 | 37 | 5 | 0.7 | 1.2 | Positive |
| C12 | No | 344.3 | 85.1 | 166 | 44 | 5 | 1.1 | 1.2 | Positive |
| C12:1 | No | 342.2 | 85.1 | 166 | 44 | 5 | 1.2 | 1.2 | Positive |
| C14 | No | 372.3 | 85.1 | 166 | 45 | 5 | 1.35 | 1.2 | Positive |
| C14:1 | No | 370.3 | 85.1 | 166 | 45 | 5 | 1.1 | 1.2 | Positive |
| C16 | No | 400.2 | 85.1 | 166 | 51 | 5 | 1.85 | 1.2 | Positive |
| C16:1 | No | 398.3 | 85.1 | 166 | 51 | 5 | 1.5 | 1.2 | Positive |
| C16-OH | No | 416.3 | 85.1 | 166 | 54 | 5 | 1.65 | 1.5 | Positive |
| C18 | No | 428.4 | 85.1 | 166 | 60 | 5 | 2.4 | 1.2 | Positive |
| C18:1 | No | 426.3 | 85.1 | 166 | 55 | 5 | 2 | 1.2 | Positive |
| C18:2 | No | 424.3 | 85.1 | 166 | 54 | 5 | 1.8 | 1.2 | Positive |
| C2 | No | 204.2 | 85.1 | 166 | 20 | 5 | 0.6 | 1.2 | Positive |
| C3 | No | 218.4 | 85.1 | 166 | 29 | 5 | 0.6 | 1.2 | Positive |
| C4 | No | 232.2 | 85.1 | 166 | 29 | 5 | 0.7 | 1.2 | Positive |
| C5 | No | 246.3 | 85.1 | 166 | 27 | 5 | 0.7 | 1.2 | Positive |
| C6 | No | 260.2 | 85.1 | 166 | 27 | 5 | 0.7 | 1.2 | Positive |
| C8 | No | 288.1 | 85.1 | 166 | 33 | 5 | 0.7 | 1.2 | Positive |
| d3C16 | Yes | 403.2 | 85.1 | 166 | 51 | 5 | 1.85 | 1.2 | Positive |
| d3C2 | Yes | 207.2 | 85.1 | 166 | 20 | 5 | 0.6 | 1.2 | Positive |
| d3C3 | Yes | 221.4 | 85.1 | 166 | 29 | 5 | 0.6 | 1.2 | Positive |
| d3C4 | Yes | 235.2 | 85.1 | 166 | 29 | 5 | 0.7 | 1.2 | Positive |
| d3C8 | Yes | 291.1 | 85.1 | 166 | 33 | 5 | 0.7 | 1.2 | Positive |
| d9C0 | Yes | 171.1 | 85.1 | 166 | 23 | 5 | 0.58 | 1.2 | Positive |
| d9C14 | Yes | 381.3 | 85.1 | 166 | 45 | 5 | 1.6 | 1.2 | Positive |
| d9C5 | Yes | 255.3 | 85.1 | 166 | 27 | 5 | 0.7 | 1.2 | Positive |
